# L-DOPA improves extinction memory retrieval after successful fear extinction

**DOI:** 10.1007/s00213-019-05301-4

**Published:** 2019-06-26

**Authors:** A. M. V. Gerlicher, O. Tüscher, R. Kalisch

**Affiliations:** 1 Neuroimaging Center (NIC), Focus Program Translational Neuroscience (FTN), Johannes Gutenberg University Medical Center, Langenbeckstr. 1, 55131 Mainz, Germany; 2 Deutsches Resilienz Zentrum (DRZ), Johannes Gutenberg University Medical Center, Untere Zahlbacher Str. 8, 55131 Mainz, Germany; 3 Present Address: Department of Clinical Psychology, University of Amsterdam, Nieuwe Achtergracht 129B, 1018 WS Amsterdam, The Netherlands; 4 Department of Psychiatry and Psychotherapy, Johannes Gutenberg University Medical Center, Untere Zahlbacher Str. 8, 55131 Mainz, Germany

**Keywords:** Dopamine, Fear conditioning, Extinction, Memory consolidation, Anxiety, Post-traumatic stress disorder, Exposure treatment, Cognitive-behavioural therapy

## Abstract

**Rationale:**

A promising strategy to prevent a return of fear after exposure-based therapy in anxiety disorders is to pharmacologically enhance the extinction memory consolidation presumed to occur after exposure. Accumulating evidence suggests that the effect of a number of pharmacological consolidation enhancers depends on a successful fear reduction during exposure. Here, we employed the dopamine precursor L-DOPA to clarify whether its documented potential to enhance extinction memory consolidation is dependent on successful fear extinction.

**Methods:**

In two double-blind, randomized and placebo-controlled experiments (experiment 1: *N* = 79, experiment 2: *N* = 32) comprising fear conditioning (day 1), extinction followed by administration of 150 mg L-DOPA or placebo (day 2) and a memory test (day 3) in healthy male adults, conditioned responses were assessed as differential skin conductance responses. We tested whether the effect of L-DOPA on conditioned responses at test depended on conditioned responses at the end of extinction in an experiment with a short (10 trials, experiment 1) and long (25 trials, experiment 2) extinction session.

**Results:**

In both experiments, the effect of L-DOPA was dependent on conditioned responses at the end of extinction. That is, post-extinction L-DOPA compared to placebo administration reduced conditioned responses at test only in participants showing a complete reduction of conditioned fear at the end of extinction.

**Conclusion:**

The results support the potential use of L-DOPA as a pharmacological adjunct to exposure treatment, but point towards a common boundary condition for pharmacological consolidation enhancers: a successful reduction of fear in the exposure session.

**Electronic supplementary material:**

The online version of this article (10.1007/s00213-019-05301-4) contains supplementary material, which is available to authorized users.

## Introduction

Exposure-based treatments are rooted in the principles of fear extinction and are an initially very effective intervention to reduce pathological fear and anxiety in the context of cognitive-behavioural therapy (Beck et al. 2005). In exposure-based treatments, patients are asked to confront themselves with the threat-associated stimulus or situation. The experience that no actual harm occurs during the exposure to the feared stimulus or situation results in a reduction of pathological fear and anxiety (Craske et al. 2008; Craske et al. 2014). Similarly, during fear extinction in the laboratory a conditioned stimulus (CS) that was previously paired with an aversive unconditioned stimulus (US) is repeatedly presented in the absence of the US, resulting in a reduction of conditioned fear responses (CRs). In both laboratory settings and in clinical practice CRs can, however, be re-evoked despite successful extinction learning (Bouton 2004). The occurrence of a return of fear after extinction indicates that extinction learning does not erase the initial ‘CS-US’ association (fear memory), but rather creates a competing ‘CS-noUS’ association, or extinction memory, that is suggested to inhibit the expression of fear (Bouton 2004). Thus, in order to ensure a reliable long-term reduction of fear after extinction learning or exposure treatment, the extinction memory trace has to be reliably retrieved upon a new encounter with the CS despite competition with the fear memory trace.

One strategy to facilitate extinction over fear memory retrieval is to enhance the strength of extinction memories. To this end, recent studies (Fitzgerald et al. 2014; Singewald et al. 2015) have investigated pharmacological interventions targeting either extinction learning itself or the extinction memory consolidation presumed to occur in the hours after new learning (McGaugh, 2000). The NMDA receptor agonist D-cycloserine (DCS) is among the best-studied pharmacological adjuncts to exposure therapy (Otto et al. 2016). Interestingly, animal studies in which DCS was administered either before or after extinction learning point towards a potential complication in pharmacological extinction memory enhancement. Namely, DCS was only beneficial in animals exhibiting low fear at the end of extinction (Weber et al. 2007; Bouton et al. 2008; Bolkan and Lattal 2014). Experimental manipulations of extinction duration revealed that only a combination of DCS with long extinction resulted in improved extinction memory retrieval, whereas DCS administration after short extinction did either have no effect (Bouton et al. 2008) or even led to greater fear responses at test (Lee et al. 2006). It was suggested that a short extinction session during which CRs remain high may merely evoke a reactivation of the fear memory trace (Merlo et al. 2014). Upon reactivation, fear memories become malleable and labile again. For memories to persist after and beyond reactivation, they must be stabilized by fear memory *reconsolidation* processes (Nader et al. 2000). Administering DCS after short extinction may, thus, have enhanced the fear memory reconsolidation and strengthened the fear memory. In contrast, during a long extinction session during which CRs are completely reduced, an extinction memory trace may have been acquired successfully and a subsequent DCS administration may have enhanced extinction memory consolidation as desired. Thus, the behavioural expression of fear at the end of an extinction session may be an indicator of the currently active memory trace that is susceptible to pharmacological enhancement (Eisenberg et al. 2003; King et al. 2018). Analogous observations have been made in patient studies combining exposure treatment with DCS (Smits et al., 2013a, b; but see de Kleine et al. 2015), the α2-adrenergic agonist yohimbine (Smits et al. 2014) or the neuro-metabolic enhancer methylene blue (Telch et al. 2014) across different anxiety disorders. Also in patients, the effects of DCS, yohimbine or methylene blue were a function of subjective fear at the end of exposure.

Together with others, we recently introduced L-DOPA, a dopamine precursor, as a new enhancer of extinction memory consolidation. Post-extinction L-DOPA administration in rodents improved extinction memory retrieval, reducing the return of fear after the mere passage of time (‘spontaneous recovery’), after an unannounced re-confrontation with the US (‘reinstatement’) and even after confrontation with the CS in a context different from the extinction context (Haaker et al. 2013; Whittle et al. 2016), a phenomenon called ‘contextual renewal of fear’. In two MRI studies, we translated these findings to humans (Haaker et al. 2013; Gerlicher et al. 2018) and identified a mechanism via which dopamine enhances human extinction memory consolidation. Namely, post-extinction L-DOPA administration increased spontaneous reactivations of an extinction learning-related prefrontal activity pattern in the hours after extinction learning (Gerlicher et al. 2018). Interestingly, in these data, we also observed a weak relationship between conditioned responses at the end of extinction and extinction memory retrieval after L-DOPA intake (Gerlicher et al. 2018). Even though only trend-wise significant, this observation indicates that—similar to DCS, yohimbine and methylene blue—memory consolidation enhancement by L-DOPA may also depend on a successful reduction of fear at the end of extinction.

**Fig. 1:**
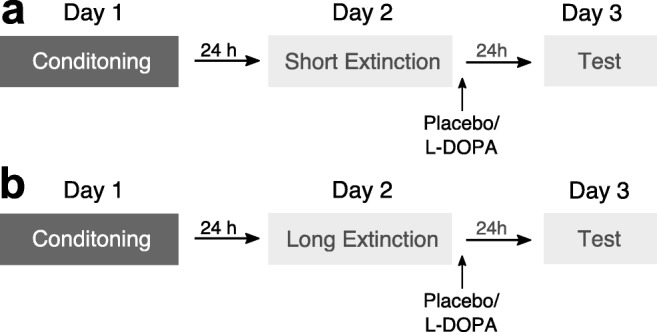
Experimental design of experiment 1 and 2. **a** In order to test the potential influence of inter-individual differences in extinction success on the effect of a post-extinction administration of L-DOPA on extinction memory retrieval, we aimed to increase inter-individual variability in extinction success by reducing the number of trials during extinction compared to a previous study (Gerlicher et al. 2018) from 15 to 10 CS+ and CS- trials in experiment 1. **b** In experiment 2, we aimed to induce successful extinction learning experimentally by increasing the number of trials during the extinction session from 15 in a previous study (Gerlicher et al. 2018) to 25 CS+ and CS- trials (long extinction) in experiment 2

Here, we aimed to clarify whether the effect of a post-extinction L-DOPA administration is indeed dependent on extinction success. We conducted two experiments with conditioning (in context A) on day 1, extinction (in context B) and subsequent administration of either L-DOPA or placebo on day 2 and a test of extinction memory retrieval (in the extinction context B) on day 3. In experiment 1, a short extinction session (10 trials) was employed with the intention to assure sufficient inter-individual variability in CRs at the end of extinction due to incomplete extinction learning in some subjects but not in others (Fig. 1a). Given high inter-individual variability in extinction success, we expected to find that the effect of L-DOPA on conditioned fear at test would be dependent on conditioned fear responses at the end of extinction. In experiment 2, we aimed to extend the correlative evidence from experiment 1 by increasing the number of extinction trials (long extinction, 25 trials) in order to produce low differential CRs at the end of extinction on day 2 consistently across participants (Fig. 1b). We hypothesised that after successful extinction a post-extinction L-DOPA compared to placebo administration would significantly reduce conditioned fear at test. In line with our previous studies (Haaker et al. 2013; Haaker et al. 2015; Gerlicher et al. 2018), we assessed conditioned responses (CRs) as skin conductance response (SCR) to the reinforced CS+ compared to an unreinforced CS-. With regard to the commonly reported differential effects of pharmacological memory manipulations on different measures of conditioned fear in humans (Kindt et al. 2009; Soeter and Kindt 2010, 2011, 2012; Sevenster et al. 2012, 2014), we also assessed fear-potentiated startle responses (FPS) and online fear ratings to CS+ and CS- as secondary outcome measures.

## Materials and methods

### Participants

In total, 80 healthy male participants took part in experiment 1. One participant had to be excluded due to recording software problems, resulting in a sample size of 79 (mean ±SD age, 27 ± 2 years) in experiment 1. In experiment 2, we tested 32 (mean ± SD age, 28 ± 3 years) healthy male participants. In experiment 2, the required sample size was estimated based on the effect of L-DOPA on differential SCR at test in successful extinguishers in experiment 1 using G*Power (Faul et al. 2007; stim*group partial *η*^*2*^ = .29, power 1 − β = .80, correlation among repeated measures *r* = .50) and further increased in order to compensate for the low signal-to-noise ratio of psychophysiological measures. We restricted recruitment to male participants as the estrous cycle interacts with extinction memory consolidation (Lebron-Milad and Milad 2012; Cover et al. 2014) and dopamine can have opposing effects on extinction depending on estrous cycle phase (Rey et al. 2014). A board-certified physician screened participants for contraindications of L-DOPA intake, current physiological, neurological or psychiatric disorders, excessive consumption of nicotine (> 10 cigarettes/day), alcohol (> 15 glasses of beer/wine per week) or cannabis (> 1 joint/month), participation in other pharmacological studies and tinnitus (as contraindication for startle probe exposure). Drug abuse was assessed via urine test (M-10/3DT; Diagnostik Nord, Schwerin, Germany). During the screening session, we also tested skin conductance responding in each participant. To this aim, we attached two electrodes of the eSense skin response device (Mindfield® Biosystems Ltd., Berlin, Germany) to the medial phalanges of the index and middle finger. Participants were asked to take several deep breaths. In addition, the physician clapped his hands without announcement. Both deep breathing and acoustic startle usually result in a deflection of the skin conductance, not seen in skin conductance non-responding individuals. None of the participants screened for the present experiments had to be excluded according to this criterion. Both experiments were approved by the local ethics committee (Ethikkommission der Landesärztekammer, Rhineland-Palatinate, Germany) and conducted in accordance with the Declaration of Helsinki.

### Stimuli

Two black geometric symbols (triangle, circle) served as CSs. The CSs were presented in the centre of a computer screen and super-imposed on background pictures of either a kitchen or a living room, which served as context A or B. Assignment of symbols to CS+/CS- and rooms to contexts A/B was counter-balanced between participants and groups. A painful electrical stimulation consisting of three square-wave pulses (50 ms inter-stimulus interval) of 2 ms was employed as US. Pain stimuli were generated by a DS7A electrical stimulator (Digitimer Ltd., Welwyn Garden City, UK) and delivered on the right dorsal hand through a surface electrode with platinum pin (Specialty Developments, Bexley, UK).

### Experimental procedure

#### Day 1—conditioning

Upon arrival, participants completed questionnaires on trait and state anxiety (STAI-T/STAI-S; Spielberger et al. 1970), anxiety sensitivity (ASI-3; Taylor et al. 2007) and demographic data. Subsequently, electrodes were attached and US intensity was calibrated to a level rated as ‘maximally painful, but still tolerable’. Familiarization consisted of two CS presentations in both contexts and a practice rating of fear and US expectancy. Before the start of the actual experiment, participants were instructed that the experiment would be distributed across 3 days, that one symbol would never be followed by an electric shock and that their task was to find out what rule applied to the other symbol. The experiment started every day with US expectancy ratings for each CS, followed by a habituation phase. During the habituation phase participants were presented with 30 s background noise and 10 startle probes (noise alone trials, NA), during which the context was already presented on the computer screen. The context remained on the screen continuously throughout the experiment. Participants were asked to rate their subjective fear/distress/tension within the first 5 s of the CS presentation. The CS was presented for 8 s in total. Startle probes were delivered 7.4 s after CS onset. In case of reinforced CS+ presentations, USs were delivered 500 ms later. Inter-trial intervals (ITI) lasted 15, 20 or 25 s (mean of 20 s). Trial order was randomized and not more than two trials of the same type (i.e. CS+, CS-, NA) succeeded each other. During conditioning, participants were presented with 5 CS+, 5 CS- and 5 NA trials in context A in both experiments 1 and 2. In both experiments, four out of 5 CS+ presentations (i.e. 80%) were reinforced. After conditioning, participants again rated their US expectancy. The total duration of the session amounted to approximately 1 h on day 1.

#### Day 2—extinction

The extinction session took place approximately 24 h (± 1 h) after conditioning. Upon arrival participants were asked to fill out the STAI-S. We did not re-calibrate the US, but informed participants that their individual US strength from day 1 would be applied and that the experiment would continue. In experiment 1, the extinction session consisted of 10 CS+, 10 CS- and 10 NA trials presented in context B. In experiment 2, the extinction session consisted of 25 CS+, 25 CS- and 25 NA trials presented in context B. After the extinction session electrodes were detached and participants were administered either a placebo or L-DOPA pill. After pill intake, participants remained under observation for 1 h while blood pressure and heart rate were monitored regularly. Participants then filled out an L-DOPA side effects questionnaire and the STAI-S. The total duration of the session amounted to approximately 2.5 h on day 2.

#### Day 3—test

The test session took place approximately 24 h (± 1 h) after the extinction session. Upon arrival, participants were asked to fill out the side effects questionnaire and the STAI-S. After electrode attachment, participants were again only instructed that the experiment would continue and presented with 8 CS+, 8 CS- and 8 NA trials in context B in both experiments 1 and 2. The total duration of the session amounted to approximately 45 min on day 3.

### Drug treatment

Participants were asked to refrain from eating, drinking and smoking for 2 h prior to drug intake. Participants were randomly assigned to the L-DOPA or placebo group with the restriction that groups were matched on STAI-T and ASI scores. Assignment of participants to the placebo or L-DOPA group was conducted by a person not involved in data collection. Participants were either administered 150/37.5 mg levodopa-benserazide (Levodopa-Benserazid-ratiopharm®, Germany; for dosage, see (Haaker et al. 2013, 2015) or an identically looking capsule filled with mannitol and aerosol (placebo). Drugs were prepared and provided by the pharmacy of the University Medical Center Mainz and administered double-blind.

### Skin conductance response

Electrodermal activity was recorded from the thenar and hypothenar of the left hand using self-adhesive Ag/AgCl electrodes (EL-509, BIOPAC® Systems Inc., Goleta, CA, USA). The raw signal was amplified and low-pass filtered with a cut-off frequency of 1 Hz. Using a custom-made analysis script, we manually scored the first local minimum in the skin conductance time course in a time window from 900 to 4000 ms after CS onset as response onset (Boucsein et al. 2012) and the following local maximum as response peak (onset to peak latency 500–4000 ms). We assessed the amplitude of an SCR then as onset-to-peak difference. Critically, the experimenter scoring the data was blinded to both stimulus type (CS+/CS-) of each SCR and group belongingness (placebo/L-DOPA) of each participant. Responses smaller than 0.02 μs were scored as zero and remained in the analysis. If more than 75% of trials had to be scored as zero, data of that subject/day was considered invalid. In experiment 1, this applied to *n* = 3/2/6 (day 1/2/3) participants, in experiment 2 to *n* = 1/0/2 participants, leaving *N* = 35/35 L-DOPA/placebo complete data sets in experiment 1 and *N* = 16/14 L-DOPA/placebo data sets in experiment 2 for statistical analysis. To normalize distributions, data were log-transformed (after a constant of 1 was added) and range-corrected within subject and day (Lykken and Venables 1971).

### Fear-potentiated startle response

The eye blink reflex was elicited by a loud noise (40 ms/104 dB) presented against constant broadband noise (70 dB) via headphones (Sennheiser, HD 380 pro). Two 7-mm Ag/AgCl electrodes filled with electrolyte gel (Signa Gel, Parker) were positioned approximately 1 cm below the pupil and 1 cm below the lateral canthus, the outer corner of the right eye (Fridlund and Cacioppo 1986). We recorded the electromyographic signal using the BIOPAC MP150 and the EMG100C device. The signal was amplified and band-pass filtered between 10 and 500 Hz and offline rectified and integrated with a time constant of 10 ms using a custom-made analysis script. FPS amplitudes were scored as the difference between response onset and maximum of responses elicited within 20–120 ms after startle probe onset. If a session comprised more than 75% of trials affected by artefacts or blinks, data from that subject/day was considered invalid. This applied to *N* = 8/2/7 (day 1/2/3) participants in experiment 1 and *N* = 1 participant on day 3 in experiment 2, leaving *N* = 31/32 L-DOPA/placebo (experiment 1) and *N* = 15/16 L-DOPA/placebo (experiment 2) complete data sets for statistical analysis. Data of each participant were standardized per day (*z*-score) and linearly transformed to *T*-scores.

### Fear and US expectancy ratings

Within the first 5 s of each CS presentation participants were asked to indicate their fear/distress/tension on a visual analogue scale ranging from 0 = ‘no fear/distress/tension’ to 100 = ‘high fear/distress/tension’ with a mouse-button click. If participants did not respond within this time window, missing values were replaced by subject- and day-specific means. Before and after each experimental phase, participants were asked to indicate the expectancy of receiving an electric shock for each CS on a scale from 0 = ‘no expectancy’ to 100 = ‘high expectancy’.

### Statistical analysis

**Fig. 2:**
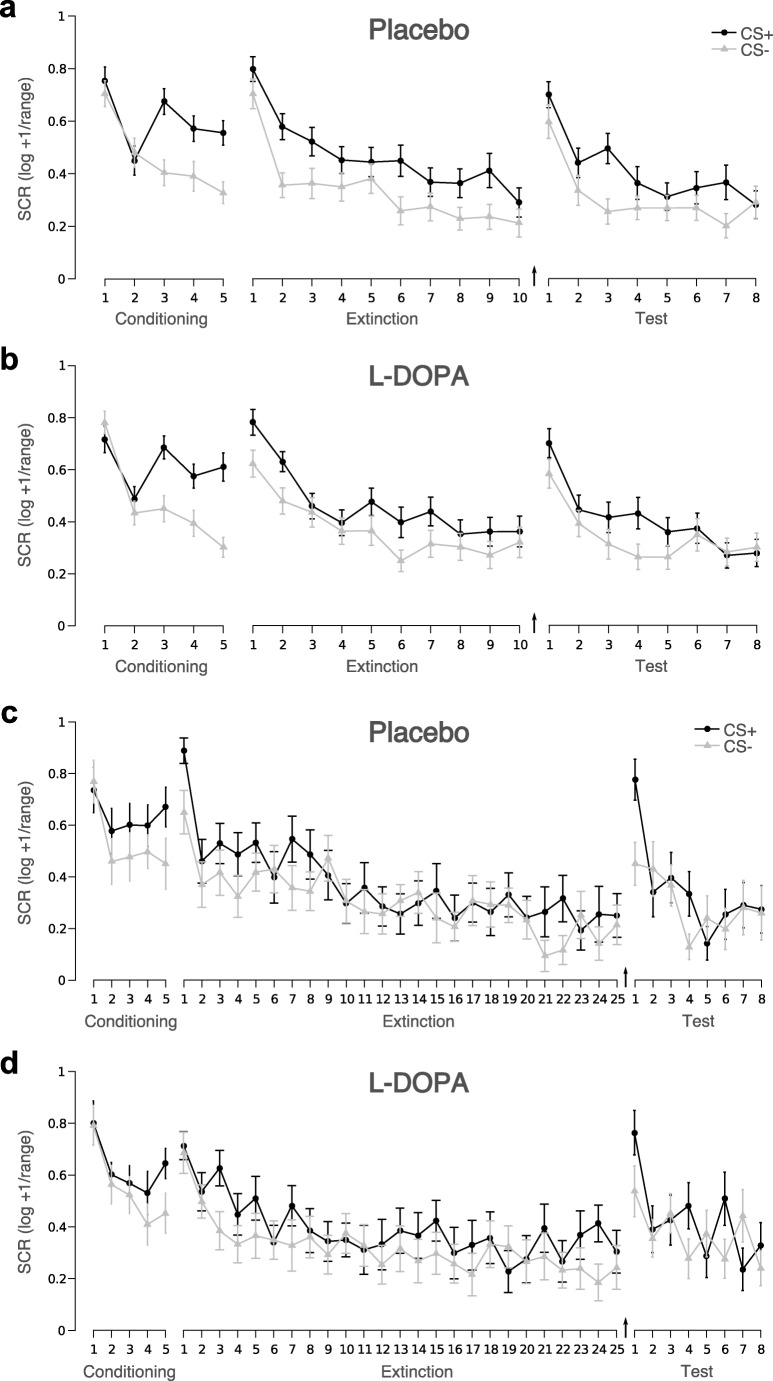
Trial by trial SCRs to CS+ and CS- during conditioning on day 1, extinction on day 2 and test on day 3. Skin conductance responses of **a** placebo (*N* = 35) and **b** L-DOPA-treated (*N* = 35) participants in experiment 1 did not differ significantly in any of the experimental phases. In contrast to previous findings (Gerlicher et al. 2018), there was no of effect of L-DOPA administration after short extinction on group average CS+ compared to CS- SCRs during test on day 3. Similarly, in experiment 2 groups treated with **c** placebo (*N* = 14) and **d** L-DOPA (*N* = 16) after a long extinction session did also not differ significantly in any of the experimental phases. Note, however, that the intended manipulation of prolonging extinction in order to fully reduce CRs was not successful and the long extinction session did not result in a complete reduction of differential (CS+ > CS-) SCRs at the end of the extinction session. Error bars depict standard error of the mean. Arrows indicate time point of drug administration

For statistical analysis of experimental and group effects on SCRs, we operationalized CRs at the end of conditioning (i.e. two trials in experiment 1 and 2) and CRs at the beginning or end (i.e. two trials in experiment 1, five trials in experiment 2) of extinction, by averaging SCRs to CS+ and CS- over 20% of trials at the beginning or end of the respective experimental phase. This procedure was a-priori standardized with the aim to harmonize measures across experiments with varying trial numbers (Gerlicher et al. 2018). As in previous studies (Haaker et al. 2013, 2015; Gerlicher et al. 2018), the effect of L-DOPA on extinction memory retrieval was tested on SCRs elicited by CS+ and CS- averaged across the whole test phase on day 3 (i.e. eight trials in both experiments 1 and 2). These a-priori determined operating procedures are an attempt to prevent post hoc biases in operationalization decisions (Ney et al. 2018). Please see Fig. 2 for single-trial SCRs in both experiments.

Repeated measures ANOVA with stimulus (CS+/CS-) as within- and group (placebo/L-DOPA) as between-subject factor was employed to assess whether the placebo and L-DOPA group differed in CRs at the end of conditioning, the beginning or the end of extinction and to test the potential effect of post-extinction L-DOPA administration on CRs at test on day 3 on SCR data. To specifically test the hypothesis that the effect of L-DOPA on conditioned fear at test on day 3 was dependent conditioned fear at the end of extinction on day 2, multiple linear regression analysis was performed with differential CRs (SCR CS+>CS-) at the end of extinction, group (placebo/L-DOPA) and their interaction as independent and differential CRs (SCR CS+>CS-) at test as dependent variable. We also included differential CRs at the end of conditioning and the beginning of extinction as covariates into the regression analysis as extinction success could have been confounded by initial fear acquisition or fear at the beginning of extinction.

Statistical analysis of FPS, online fear and US expectancy rating data followed the same procedure (for results see Supplementary Fig. 1–6). All statistical tests were conducted two-sided and considered significant when *P* < .05.

#### Availability of data and material

All data and material presented in this article are available online:

https://osf.io/keqrn/?view_only=459c7faa579748c294b876cec64c3570.

## Results

### Experiment 1

Participants in the L-DOPA and placebo groups did not differ significantly on STAI-T, STAI-S and ASI scores, US amplitude, US rating or reported side effects (independent sample *t* tests, all *p* > .12: see Supplementary Table 1). A repeated measures ANOVA with stimulus as within- and group as between-subject factor showed that groups did not differ on CRs at the end of conditioning on day 1 (stim: *F*(1,68) = 57.95, *p* < .001, partial *η*^*2*^ = .46; group: *F*(1,68) = .05, *p* = .83; stim*group: *F*(1,68) = .47, *p* = .49). Groups did also not differ on CRs at the beginning (stim: *F*(1,68) = 20.99, *p* < .001, partial *η*^*2*^ = .24; group: *F*(1,68) = .25, *p* = .62; stim*group: *F*(1,68) = .00, *p* = .97) or end (stim: *F*(1,68) = 12.73, *p* = .001, partial *η*^*2*^ = .16; group: *F*(1,68) = .48, *p* = .49; stim*group: *F*(1,68) = 1.23, *p* = .25) of extinction on day 2. Notably, post-extinction L-DOPA administration after the short extinction session in experiment 1 did not reduce CRs at test on day 3 (Fig. 2a, b; stim: *F*(1,68) = 16.80, *p* < .001, partial *η*^*2*^ = .20; group: *F*(1,68) = .12, *p* = .74; stim*group: *F*(1,68) = .77, *p* = .38). Similar results were obtained for FPS (Supplementary Fig. 1), online fear ratings (Supplementary Fig. 2) and pre- and post-phase US expectancy ratings on all days (Supplementary Fig. 3).

**Fig. 3:**
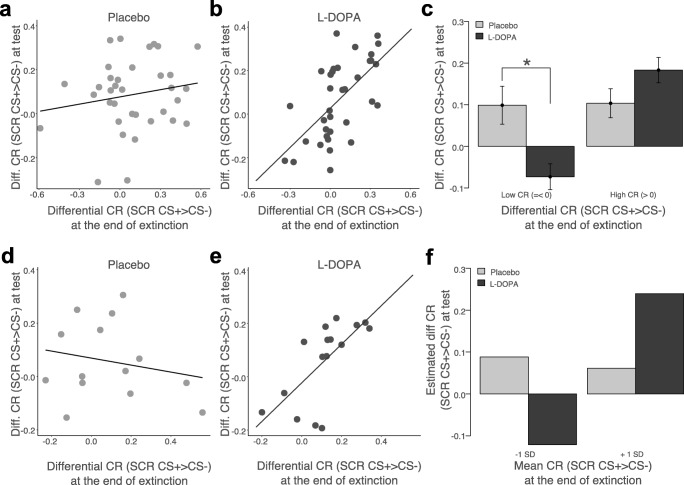
Relationship between CRs at the end of extinction on day 2 and CRs during test on day 3 in placebo and L-DOPA-treated participants in experiments 1 (upper panels) and 2 (lower panels). **a** Whereas there was no significant relationship between CRs (SCR CS+ > CS-) at the end of extinction on day 2 and CRs (SCR CS+ > CS-) at test on day 3 in placebo-treated participants (*N* = 35), **b** there was a significant positive relationship after post-extinction L-DOPA administration (*N* = 35) in experiment 1. **c** Post hoc comparison of L-DOPA (*N* = 16) compared to placebo-treated individuals (*N* = 12) with a complete reduction of CRs ((SCR CS+ > CS-) ≤ 0, ‘low CR’ in the panel) revealed that L-DOPA significantly improved extinction memory retrieval after successful within-session extinction. In contrast L-DOPA (*N* = 19) compared to placebo-treated (*N* = 23) individuals with high CRs ((SCR CS+ > CS-) > 0) at the end of extinction showed a trend towards impaired extinction memory retrieval. **d** Replicating the effect of experiment 1, also in experiment 2 not placebo (*N* = 14), but **e** only L-DOPA-treated participants (*N* = 16) showed a significant positive relationship between CRs (SCR CS+ > CS-) at the end of extinction on day 2 and CRs (SCR CS+ > CS-) at test at test 24 h later. **f** Point estimates of CRs (SCR CS+ > CS-) at test for participants with low (mean – 1 SD) and high (mean + 1 SD) CR at the end of extinction show that also in experiment 2, L-DOPA was only beneficial in individuals showing successful within-session extinction, but may have detrimental effects after non-complete within-session extinction. Error bars depict standard error of the mean

However, in line with the hypothesis that extinction success determines the effect of L-DOPA, multiple regression analysis revealed a significant interaction between CRs at the end of extinction learning and group (*β*_endfear*group_ = .45, SE = .18, *t*(64) = 2.69, *p* = .009; Fig. 3a, b). We employed analysis of simple slopes (cf. Aiken and West 1991) that takes data from all participants into account to probe the nature of this interaction. As visible in Fig. 3b and confirmed by simple slope analysis, differential CRs at the end of extinction and differential CRs at test were significantly positively related in L-DOPA-treated participants (*β*_endfear_ = .60, SE = .15, *t*(64) = 4.13, *p* < .001). That is, CRs at test were low in L-DOPA-treated participants with low CRs at the end of extinction, but high in L-DOPA-treated participants with high CRs at the end of extinction. Critically, there was no relationship between CRs at the end of extinction and test in the placebo group (*β*_endfear_ = .12, SE = .11, *t*(64) = 1.13, *p* = .26; Fig. 3a), indicating that CRs at the end of extinction do not predict CRs at test under placebo (normal) conditions.

Interestingly, there was no group-specific relation between CRs at the end of extinction and CRs at test as assessed by FPS or fear ratings (FPS: *β*_endfear*group_ = − .12, SE = .16, *t*(57) = − .75, *p* = .46; fear ratings: *β*_endfear*group_ = − .05, SE = .11, *t*(73) = − .46, *p* = .65).

In all regression analyses, omitting CRs at the end of conditioning on day 1 and CRs at the beginning of extinction on day 2 as covariates did not change the results (data not shown). Additional exploratory analyses trying to predict CRs at test from the difference between CRs at the beginning and end of extinction did not reveal any significant relation in either treatment group (*β*_delta*group_ = − .21, SE = .12, *t*(66) = − 1.67, *p* = .10), suggesting that the relative reduction of CRs from the beginning to the end of extinction (i.e. within-session habituation) might be a comparatively less important determinant of the L-DOPA effect compared to absolute levels of fear responding at the end of extinction.

In order to exclude that inter-individual differences in differential CRs at the end of extinction were the result of differences in US magnitude, perceived US intensity, initial fear acquisition, start-fear, STAI-T, ASI or day 2 STAI-S scores, we directly correlated extinction end-fear with these measures. However, neither of the measures showed a significant relation to extinction end-fear (all |*r*|’s < .21, all *p*’s > .08, not corrected for multiple tests).

### Experiment 2

Relative to experiment 1, we had prolonged extinction learning from 10 to 25 trials in experiment 2 in order to achieve low CRs at the end of extinction in all subjects. In experiment 2, placebo and L-DOPA groups did also not differ significantly in respect to STAI-T, STAI-S, ASI scores, US amplitude, US rating or reported side effects (all *t* < .81, all *p* > .43; see Supplementary Table 2). Repeated measures ANOVA confirmed that groups did not differ in CRs at the end of conditioning on day 1 (stim: *F*(1,28) = 13.99, *p* = .001, partial *η*^2^ = .33; group: *F*(1,28) = .32, *p* = .57; stim*group: *F*(1,28) = .00, *p* = .97) or CRs at the beginning of extinction (stim: *F*(1,28) = 37.71, *p* < .001, partial *η*^2^ = .53; group: *F*(1,28) = .00, *p* = .97; stim*group: *F*(1,28) = .43, *p* = .52). However, unexpectedly, prolonged extinction learning did not lead to a complete reduction of CRs, but there was still a significant effect of stimulus at the end of extinction (stim: *F*(1,28) = 8.81, *p* = .006, partial *η*^2^ = .24) in both groups (group: *F*(1,28) = 1.40, *p* = .25; stim*group: *F*(1,28) = .09, *p* = .77). In addition, as in experiment 1, there was an absence of an unqualified (average group-level) effect of L-DOPA on CRs during test on day 3 (stim: *F*(1,28) = 4.53, *p* = .04, partial *η*^2^ = .14; group: *F*(1,28) = 1.09, *p* = .31; stim*group: *F*(1,28) = .00, *p* = .98). Similar results were obtained for FPS (Supplementary Fig. 4), online fear ratings (Supplementary Fig. 5) and pre- and post-phase US expectancy ratings during all experimental phases (Supplementary Fig. 6). We therefore applied the same analysis strategy as in experiment 1 and tested whether extinction success moderated the effect of L-DOPA on CRs at test.

Confirming the results of experiment 1, multiple regression analysis again revealed that extinction success affects the effect of L-DOPA, as indicated by a significant interaction between group and CRs at the end of extinction (*β*_endfear*group_ = 1.03, SE = .33, *t*(24) = 3.09, *p* = .005; Fig. 3d, e). Follow-up analysis of simple slopes (cf. Aiken and West 1991) that takes data from all participants into account showed that CRs at the end of extinction and CRs during test were positively related in the L-DOPA group (Fig. 3e; *β*_endfear_ = .98, SE = .30, *t*(24) = 3.22, *p* = .004). That is, L-DOPA preserved low fear in participants with successful extinction and high fear in participants with non-successful fear reduction on day 2. As in experiment 1, there was no significant relationship between CRs at the end of extinction and CRs at test in the placebo group (*β*_endfear_ = − .07, SE = .16, *t*(24) = − .38, *p* = .64) (Fig. 3d).

There were again no comparable effects on FPS (*β*_endfear*group_ = − .24, SE = .36, *t*(25) = − .68, *p* = .50) and fear ratings (*β*_endfear*group_ = − .25, SE = .19, *t*(26) = − 1.29, *p* = .21), and omitting initial fear acquisition and start-fear as covariates in all regression analyses did not change the results (data not shown). The moderating effect of the difference between CRs at the beginning and end of extinction was non-significant (*β*_delta*group_ = − .66, SE = .40, *t*(26) = − 1.65, *p* = .11), confirming that within-session reduction of CRs is not a robust predictor of the effect of L-DOPA on CRs at test.

We again tested whether we could identify any predictor of successful extinction learning. However, US magnitude, US intensity ratings, STAI-T, ASI and STAI-S scores on day 2, differential CRs at the end of conditioning or the beginning of extinction, were not related to differential CRs at the end of extinction learning (all |*r*|’s < .29, all *p*’s > .12).

### The effect of L-DOPA vs. placebo in participants with successful and non-successful extinction

To further qualify the results, we finally tested whether L-DOPA is superior to placebo administration in a sub-group of participants with successful extinction only (low CR at the end of extinction: (SCR CS+ > CS-) ≤ 0; *n* = 28) in experiment 1. Repeated measures ANOVA with stimulus as within- and group as between-subject factor revealed that after a complete reduction of CRs at the end of extinction L-DOPA compared to placebo-treated participants showed significantly reduced CRs at test on day 3 (Fig. 3c; stim: *F*(1,26) = .24, *p* = .63; stim*group: *F*(1,26) = .10.47, *p* = .003, partial *η*^2^ = .29). Specifically, this effect was due to significantly smaller differential SCRs to the CS+ during test in L-DOPA compared to placebo-treated participants with successful extinction (*t*(26) = 2.08, *p* = .047, Cohen’s *d* = .80; Fig. 3f). In contrast, in participants with non-successful extinction (high CR at the end of extinction: (SCR CS+ > CS-) > 0); *n* = 42), there was no significant effect of drug administration, but there was a weak trend towards greater CRs at test after L-DOPA compared to placebo administration (Fig. 3c; stim: *F*(1,40) = 36.97, *p* < .001, partial *η*^2^ = .48; stim*group: *F*(1,40) = 2.85, *p* = .099), indicating that L-DOPA administration after non-successful extinction may even have detrimental effects on long-term expression of fear. The same results are obtained when following the approach of Aiken and West (1991) and computing regression model-based point estimates of CRs at test for different levels of CRs at the end of extinction for each drug group (Supplementary Fig. 7).

Given the small sample size in experiment 2 that prevents separate sub-group analyses in participants with successful extinction and non-successful extinction, we followed Aiken and West (1991) and computed model-based point estimates of CRs at test for L-DOPA and placebo-treated participants with successful (low CR at the end of extinction: mean − 1 SD) and non-successful (high CR at the end of extinction: mean + 1 SD) extinction. We observed the same pattern as in experiment 1: after successful extinction L-DOPA compared to placebo administration was beneficial and resulted in a significant reduction of CRs at test (Fig. 3f; *β*_group_ = − .21, SE = .08, *t*(24) = − 2.50, *p* = .02). In contrast, when administered after non-successful extinction L-DOPA was predicted to lead to significantly higher CRs at test than placebo administration (Fig. 3f; *β*_group_ = .18, SE = .07, *t*(24) = 2.38, *p* = .03).

## Discussion

In two experiments, we examined the role of inter-individual differences in extinction success on the effect of a post-extinction L-DOPA administration on extinction memory retrieval 24 h later. In both experiments 1 and 2, a post-extinction administration of L-DOPA did not result in a general reduction of conditioned fear at test. However, in line with previous findings (Smits et al. [Bibr CR51][Bibr CR52], b, 2014; Telch et al. 2014), the effect of L-DOPA on extinction memory retrieval was dependent on inter-individual differences in within-session extinction. That is, post-extinction L-DOPA administration after successful within-session extinction (low CRs at the end of extinction as assessed by differential SCRs) was associated with low CRs at test on day 3. In contrast, post-extinction L-DOPA administration after non-successful within-session extinction (high CRs at the end of extinction) was associated with high CRs at test on day 3. Critically, as previously shown (Rescorla 2006; Plendl and Wotjak 2010; Craske et al. 2014) CRs at test could not be predicted from CRs at the end of extinction in placebo-treated participants, indicating that, under normal conditions, differences in extinction memory consolidation processes occurring after extinction learning, rather than extinction learning itself, may determine whether extinction versus fear memories are retrieved.

In both experiments 1 and 2, we observed that L-DOPA administration after successful extinction results in significantly lower CRs at test than placebo administration. Thus, L-DOPA is a promising candidate as an adjunct to exposure treatments in anxiety disorders when administered after an exposure session resulting in a complete reduction of fear. In contrast, CRs at test in participants with non-successful extinction were trend-wise (experiment 1) or significantly greater (experiment 2) after L-DOPA compared to placebo administration. Thus, as with other pharmacological substances, it may be safer to only administer an extinction memory consolidation enhancing pharmacological treatment after an exposure session in which the practitioner can ensure that subjective fear is low.

If this thinking is correct, then translating a L-DOPA-based augmentation strategy to clinical practice ideally requires a reliable and objective marker for successful extinction, in order to determine a criterion that can inform the practitioner about the indication or contraindication for L-DOPA administration. In previous studies, subjective fear ratings were successfully used as a measure of extinction success in patients (Smits et al. 2013a, b, 2014; Telch et al. 2014). In contrast, in the present two experiments, subjective fear did not moderate the effect of L-DOPA. In a laboratory setting with healthy participants, fear ratings may, however, be more susceptible to  idiosyncratic rating scale usage, demand or social desirability effects than reflecting the actual emotional state of an individual. Thus, before finally rejecting subjective fear as a marker for successful extinction, their predictive validity for clinical outcomes should be explored in more detail. In addition, skin conductance measures could also be employed in a clinical setting. Ambulatory skin conductance measurements were recently shown to reflect PTSD symptom severity (Jovanovic et al. 2018) and symptom reduction after exposure treatment (Hinrichs et al. 2017). In a case study the authors further reported that skin conductance levels (SCL) reflected the outcome of a successful exposure session (Post et al, 2017). A validation of the method in larger samples is still outstanding. Together with reports of low subjective fear, a post-exposure return of SCL to a baseline SCL level, may, thus, help to reduce the risk of an inadvertent enhancement of fear memory reconsolidation by post-exposure L-DOPA administration.

A second aim of our study was to investigate whether post-extinction L-DOPA administration would also act on the expression of conditioned fear as assessed by FPS. However, in contrast to the effects of L-DOPA on SCRs in the present and other independent studies (Haaker et al. 2013; Gerlicher et al. 2018), we did not observe any effect of L-DOPA on FPS. FPS responses are thought of as a valence-specific (Hamm and Vaitl 1996) and amygdala-dependent index of conditioned fear (Davis 1992; Funayama et al. 2001; Weike et al. 2005). In contrast, SCRs are mediated by a range of different cortical and subcortical brain areas and involve the sympathetic nervous system (e.g. Dawson et al. 2007). Dissociations between SCR and FPS are commonly observed in human fear conditioning studies (Weike et al. 2005; Weike et al. 2007). Maybe due to these dissociations, many human studies investigating pharmacological manipulations of fear or extinction memory (re-)consolidation do not assess SCR and FPS simultaneously. Importantly, in those who do, differential pharmacological effects on SCR and FPS were observed, e.g. administration of the beta-adrenergic receptor blocker propranolol during fear memory reconsolidation eliminated fear as assessed by FPS, but left SCR unchanged (Kindt et al. 2009; Soeter and Kindt 2010, 2011, 2012; Sevenster et al. 2012). Understanding why a manipulation of fear memory reconsolidation affects FPS but not SCR, whereas the present manipulation of extinction memory consolidation showed the opposite effect, requires a better understanding of the locus of action of the pharmacological substances employed, the mapping of the different memory systems and the neural mediators of the behavioural fear expression measures in humans. Thus, a satisfying explanation for the differential effect of L-DOPA on SCR and FPS can presently not be provided. Importantly, though, previous reports show that changes in SCRs can precede changes in subjective fear report (Hodgson and Rachman, 1974). Similarly, a pharmacological disruption of fear memory reconsolidation was immediately reflected in improved approach behaviour at the first test after drug intake, whereas reports of reduced subjective fear only followed at subsequent tests (Soeter and Kindt, 2015). Thus, an important question for future research is whether the initial effect of LDOPA on differential SCRs is integrated across response systems after repeated tests of extinction memory retrieval.

A strong argument for employing L-DOPA over other pharmacological extinction enhancers is that it has been shown to be effective not only in improving extinction memory retrieval (Whittle et al. 2016) but also in reducing contextual renewal of fear in rodents (Haaker et al. 2013). Contextual renewal has been suggested to lie at the core of relapse of anxiety in patients (Vervliet et al. 2013) and is rarely prevented by pharmacological extinction enhancement (Singewald et al. 2015). In a previous study, we observed that L-DOPA may also prevent contextual renewal of fear in humans (Haaker et al. 2013). However, in this study, both conditioning and extinction were conducted during one experimental session, such that the effect of a post-extinction L-DOPA administration on extinction memory consolidation could not be differentiated from potential effects on fear memory consolidation. Thus, the question of whether L-DOPA also protects against renewal is still open and future studies should also include memory tests in contexts that provoke contextual renewal of fear (e.g. conditioning context or new context).

The question remains why the main effect of L-DOPA was stronger and less dependent on within-session extinction in our previous MRI studies (Haaker et al. 2013; Gerlicher et al. 2018) than in the present experiments. Several studies have observed an increase in cortisol and salivary alpha amylase (a marker of noradrenergic activity) from pre- to post-MRI scanning (Eatough et al, 2009; Muehlhan et al, 2011; van Stegeren et al, 2006; Visser et al, 2015), indicating that placing and measuring participants in an MRI scanner can lead to a release of stress hormones, even after MRI exposure on consecutive days (Lueken et al, 2012; Peters et al, 2011). As both cortisol (Merz et al, 2017) and noradrenaline (Abraham et al, 2012) can affect extinction learning and memory consolidation (but see Lonsdorf et al, 2014), the stronger main effect of L-DOPA can potentially be explained by an additive effect of cortisol/noradrenaline and L-DOPA in the MRI studies (Haaker et al. 2013; Gerlicher et al. 2018). This hypothesis is currently being tested in a pre-registered fMRI study (Hu et al. 2018).

To conclude, we here present and replicate evidence that a dopaminergic enhancement of extinction memory consolidation is successful in improving human extinction memory retrieval, but specific to conditions of low fear at the end of extinction. These results fall into line with reports about the dependence of DCS, yohimbine and methylene blue on the expression of fear at the end of exposure sessions and point towards a challenge common to pharmacological extinction memory enhancers. Our results emphasize the importance of limiting the use of extinction enhancers to exposure sessions with good fear reduction and stress the need for a reliable marker—i.e. a measure and a criterion to assess successful extinction—in order to prevent an inadvertent enhancement of fear.

## Electronic supplementary material

ESM 1 (DOCX 457 kb)
