# Supplementary material for: L-DOPA improves extinction memory retrieval after successful fear extinction
Source: Psychopharmacology (Berl). 2019 Jun 26;236(12):3401–12. doi: 10.1007/s00213-019-05301-4 (PMC6892771; doi:10.1007/s00213-019-05301-4)
Supplement: Supplementary file 1 — (DOCX 457 kb) [file 213_2019_5301_MOESM1_ESM.docx]

**Supplementary material:**

L-DOPA improves extinction memory retrieval after successful fear extinction.

A.M.V. Gerlicher^1,2,4^, O. Tüscher^2,3^, R. Kalisch^1,2^

^1^Neuroimaging Center (NIC), Focus Program Translational Neuroscience (FTN), Johannes Gutenberg University Medical Center, Langenbeckstr. 1, 55131 Mainz, Germany.

^2^Deutsches Resilienz Zentrum (DRZ), Johannes Gutenberg University Medical Center, Untere Zahlbacher Str. 8, 55131 Mainz, Germany.

^3^Department of Psychiatry and Psychotherapy, Johannes Gutenberg University Medical Center, Untere Zahlbacher Str. 8, 55131 Mainz, Germany.

^4^Present address: Department of Clinical Psychology, University of Amsterdam, Nieuwe Achtergracht 129B, 1018 WS Amsterdam, The Netherlands.

Correspondence should be addressed to A.M.V.G. (e-mail: [a.m.v.gerlicher@uva.nl](mailto:a.m.v.gerlicher@uva.nl)).

**EXPERIMENT 1**

**Supplementary Table 1.** *Questionnaire scores, BMI and US strength and rating in L-DOPA vs. placebo group in experiment 1.*

|  | L-DOPA  N = 39  mean (SD) | Placebo  N = 40  mean (SD) | t-value | p-value |
| --- | --- | --- | --- | --- |
| STAI-T | 35.6 (9.4) | 34.7 (8.9) | .44 | .66 |
| ASI-3 | 17.2 (12.0) | 15.5 (13.9) | .81 | .42 |
| STAI-S Day 1 | 35.4 (6.6) | 34.2 (8.8) | .67 | .50 |
| STAI-S Day 2 pre | 34.1 (6.9) | 32.8 (6.9) | .79 | .43 |
| STAI-S Day 2 post | 32.2 (6.8) | 31.2 (6.6) | .63 | .53 |
| STAI-S Day 3 | 33.3 (7.3) | 31.9 (8.9) | .77 | .44 |
| BMI | 23.6 (3.6) | 24.8 (3.3) | -1.57 | .12 |
| US amplitude(mA) | 11.5 (11.8) | 14.2 (9.5) | -1.11 | .27 |
| US rating (0-10) | 6.8 (1.4) | 6.7 (1.4) | .30 | .77 |

*STAI-T: trait anxiety; STAI-S: state anxiety; ASI-3: anxiety-sensitivity index; BMI: body-mass index, US rating on a scale from 0-10 (“I do not feel anything”-“strongest imaginable pain to be delivered via such an electrode”).*

**EXPERIMENT 1**

**
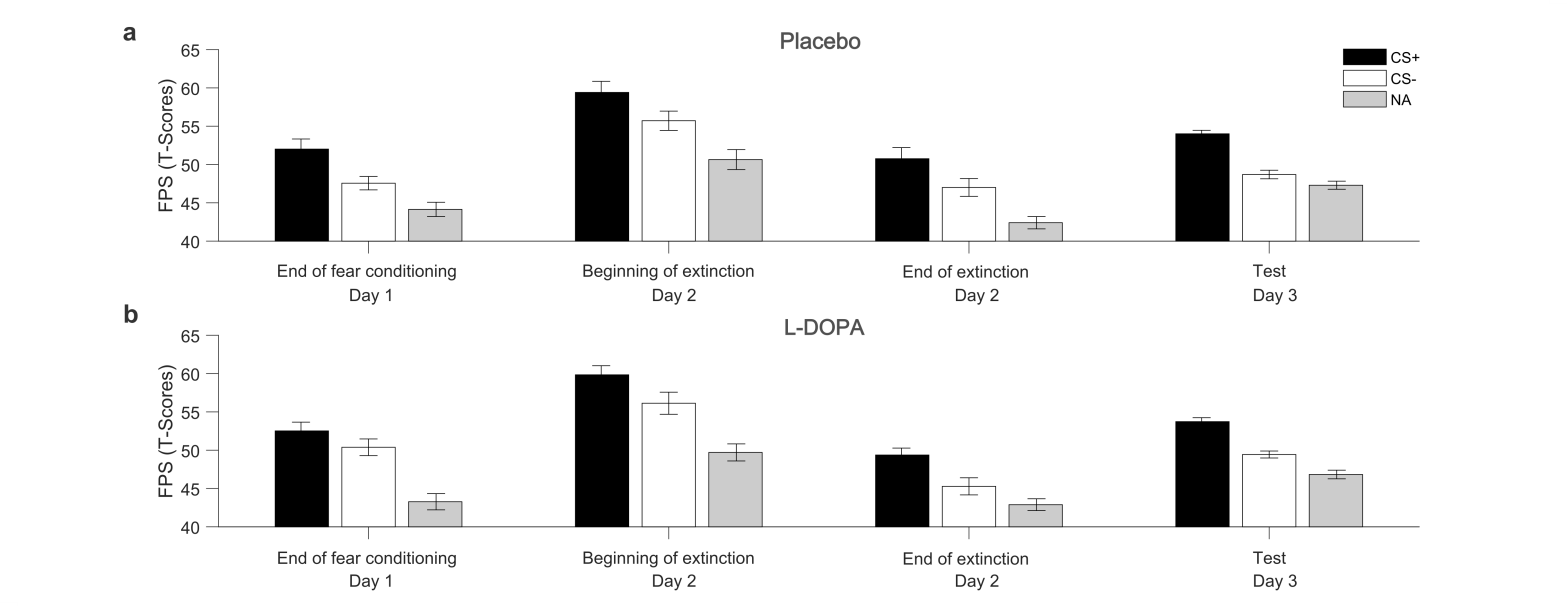
**

**Supplementary Fig. 1** Mean fear potentiated startle responses (FPS) to CS+, CS- and NA trials in experiment 1 for (a) placebo and (b) L-DOPA treated participants at the end of conditioning on day 1, at the beginning and end of extinction on day 2 and during test on day 3. Repeated measures ANOVA with stimulus (CS+/CS-) as within- and group (placebo/L-DOPA) as between-subject factor revealed no group differences in CRs as assessed by FPS at the end of conditioning on day 1 (stim: *F*(1,61)=6.60, *p*=.01, partial *η^2^*=.10; group: *F*(1,61)=3.29, *p*=.08; stim*group: *F*(1,61)=.81, *p*=.37) and the beginning (stim: *F*(1,61)=7.47, *p*=.008, partial *η^2^*=.11; group: *F*(1,61)=.10, *p*=.75; stim*group: *F*(1,61)=.00, *p*=.99) or end (stim: *F*(1,61)=11.96, *p*=.001, partial *η^2^*=.16; group: *F*(1,61)=1.61, *p*=.21; stim*group: *F*(1,61)=.02, *p*=.88) of extinction on day 2. Similar to the lack of a main effect of L-DOPA on SCRs at test on day 3 there was no group difference in CRs as assessed by FPS during test on day 3 (stim: *F*(1,61)=66.35, *p*<.001, partial *η^2^*=.52; group: *F*(1,61)=.36, *p*=.55; stim*group: *F*(1,61)=.76, *p*=.39). Error bars depict standard error of the mean.

**EXPERIMENT 1**


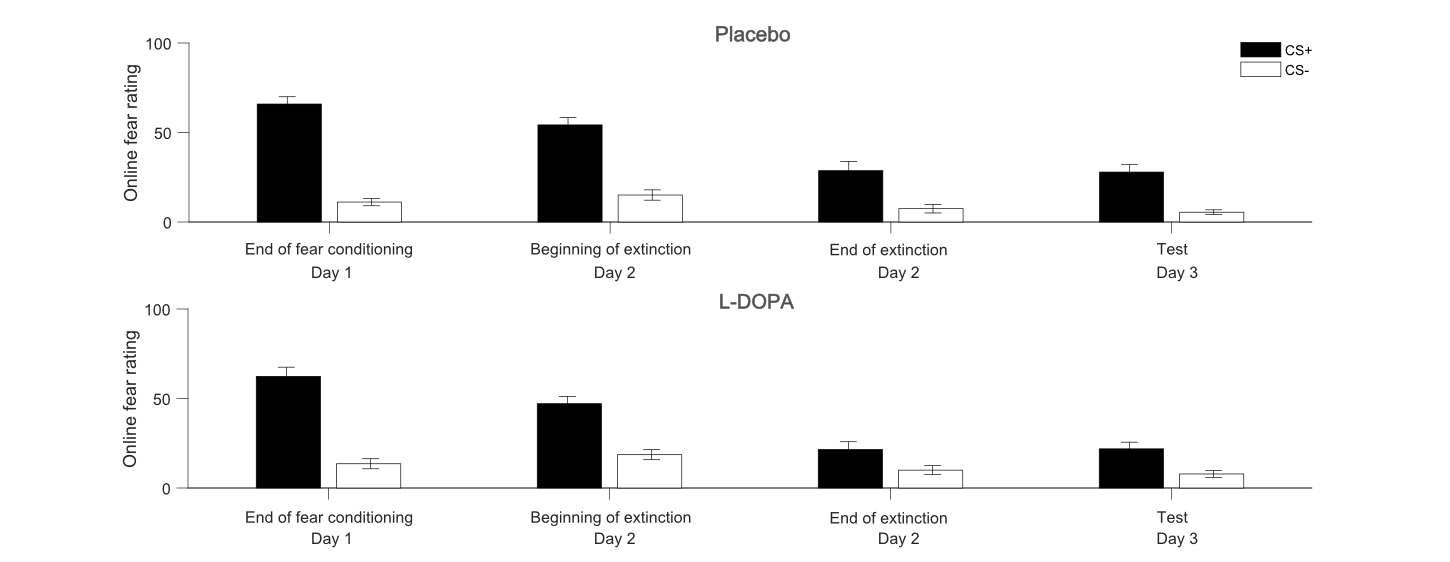


**Supplementary Fig. 2** Mean online fear ratings (0= no fear/tension/distress, 100= maximum fear/tension/distress) during CS+ and CS- presentations in experiment 1 for (a) placebo and (b) L-DOPA treated participants at the end of conditioning on day 1, at the beginning and end of extinction on day 2 and during test on day 3. Repeated measures ANOVA with stimulus (CS+/CS-) as within- and group (placebo/L-DOPA) as between-subject factor revealed no group differences in CRs as assessed by online fear ratings at the end of conditioning on day 1 (stim: *F*(1,77)=191.22, *p*<.001, partial *η^2^*=.71; group: *F*(1,77)=.02, *p*=.88; stim*group: *F*(1,77)=.63, *p*=.43) and the beginning (stim: *F*(1,77)=110.71, *p*<.001, partial *η^2^*=.59; group: *F*(1,77)=.02, *p*=.89; stim*group: *F*(1,77)=2.66, *p*=.11) or end (stim: *F*(1,77)=29.07, *p*<.001, partial *η^2^*=.27; group: *F*(1,77)=.28, *p*=.60; stim*group: *F*(1,77)=2.54, *p*=.12) of extinction on day 2. There was no effect of L-DOPA administration on online fear ratings during test on day 3 (stim: *F*(1,77)=43.55, *p*<.001, partial *η^2^*=.36; group: *F*(1,77)=.29, *p*=.59; stim*group: *F*(1,77)=2.30, *p*=.13). Error bars depict standard error of the mean.

**
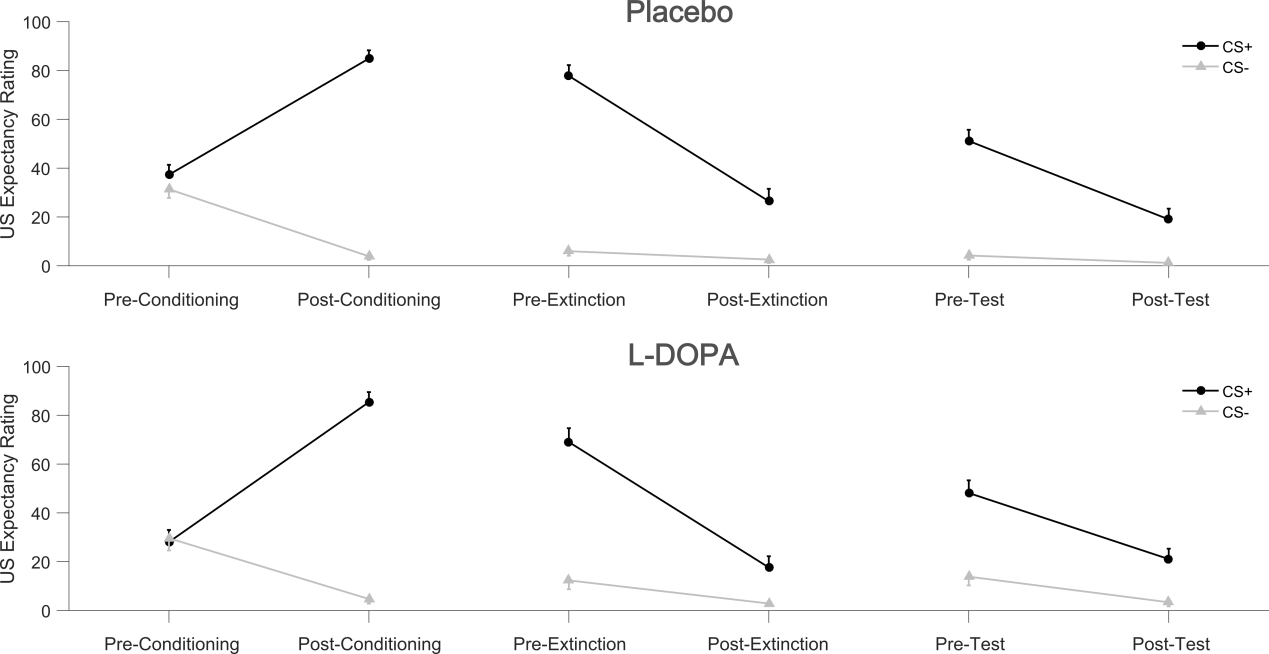
**

**Supplementary Fig. 3** Mean US expectancy ratings to CS+ and CS- in experiment 1 for (a) placebo and (b) L-DOPA treated participants collected before and after each experimental phase. Repeated measures ANOVA with stimulus (CS+/CS-) as within- and group (placebo/L-DOPA) as between-subject factor revealed no group differences in US expectancy ratings after conditioning on day 1 (stim: *F*(1,77)=676.82, *p*<.001, partial *η^2^*=.90; group: *F*(1,77)=.06, *p*=.81; stim*group: *F*(1,77)=.00, *p*=.95) or before (stim: *F*(1,77)=223.25, *p*<.001, partial *η^2^*=.74; group: *F*(1,77)=.09, *p*=.76; stim*group: *F*(1,77)=3.10, *p*=.08) or after (stim: *F*(1,77)=32.45, *p*<.001, partial *η^2^*=.30; group: *F*(1,77)=1.33, *p*=.25; stim*group: *F*(1,77)=1.81, *p*=.18) extinction on day 2. There was no significant group difference in US expectancy ratings before or after test on day 3 (stim: *F*(1,77)=118.68, *p*<.001, partial *η^2^*=.61; group: *F*(1,77)=.62, *p*=.43; stim*group: *F*(1,77)=2.88, *p*=.09). Error bars depict standard error of the mean.

**EXPERIMENT 2**

**Supplementary Table 2.** *Questionnaire scores, BMI and US strength and rating in L-DOPA vs. placebo group in experiment 2.*

|  | L-DOPA  N = 16  mean (SD) | Placebo  N = 16  mean (SD) | t -value | p-value |
| --- | --- | --- | --- | --- |
| STAI-T | 39.4 (9.8) | 42.0 (14.4) | .60 | .55 |
| ASI-3 | 18.1 (9.3) | 19.4 (10.1) | .36 | .72 |
| STAI-S Day 1 | 35.4 (8.2) | 37.5 (7.1) | -.79 | .44 |
| STAI-S Day 2 pre | 35.8 (8.8) | 36.6 (5.5) | -.34 | .74 |
| STAI-S Day 2 post | 34.2 (8.4) | 35.1 (9.6) | -.29 | .77 |
| STAI-S Day 3 | 30.8 (12.1) | 34.8 (5.7) | -1.19 | .24 |
| BMI | 24.4 (2.6) | 24.4 (3.5) | .02 | .98 |
| US amplitude(mA) | 23.3 (22.1) | 18.3 (11.7) | .81 | .43 |
| US rating (0-10) | 6.9 (1.0) | 6.8 (1.3) | .08 | .94 |

*STAI-T: trait anxiety; STAI-S: state anxiety; ASI-3: anxiety-sensitivity index; BMI: body-mass index, US rating on a scale from 0-10 (“I do not feel anything”-“strongest imaginable pain to be delivered via such an electrode”).*

**EXPERIMENT 2**

**
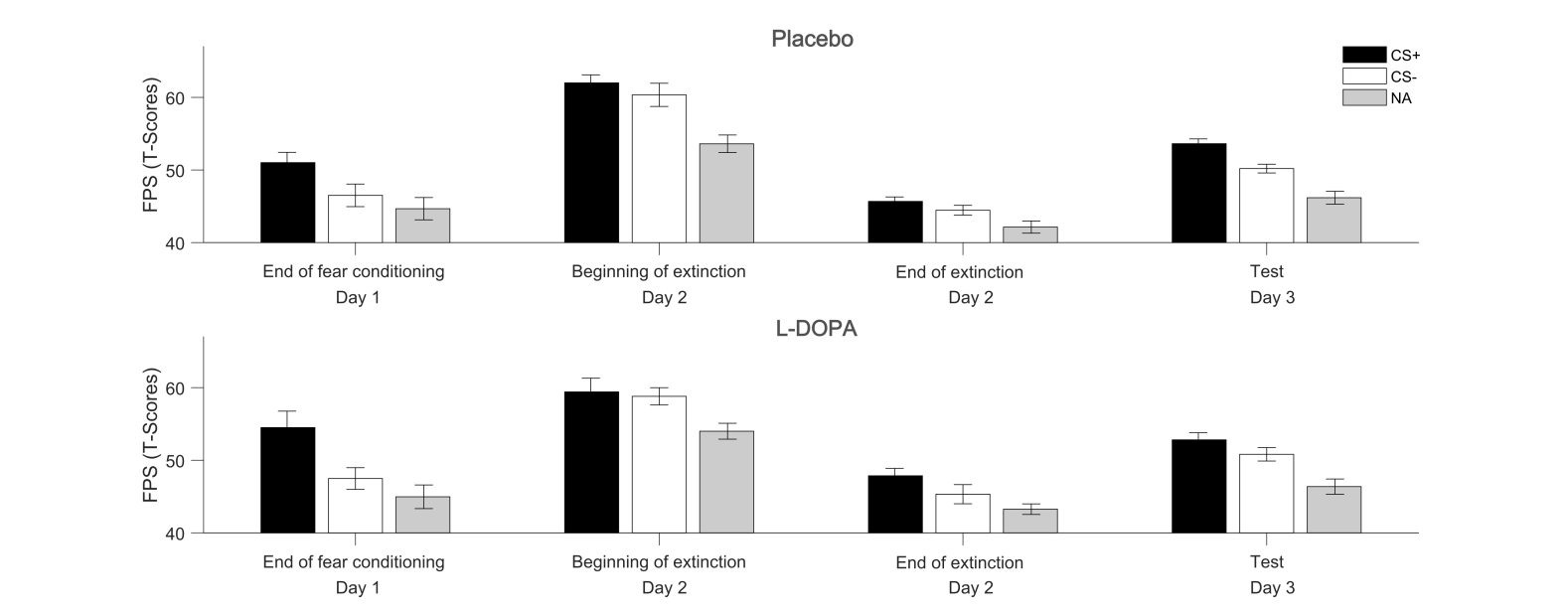
**

**Supplementary Fig. 4** Mean fear potentiated startle responses (FPS) to CS+, CS- and NA trials in experiment 2 for (a) placebo and (b) L-DOPA treated participants at the end of conditioning on day 1, at the beginning and end of extinction on day 2 and during test on day 3. Repeated measures ANOVA with stimulus (CS+/CS-) as within- and group (placebo/L-DOPA) as between-subject factor revealed no group differences in CRs as assessed by FPS at the end of conditioning on day 1 (stim: *F*(1,29)=11.82, *p*=.002, partial *η^2^*=.29; group: *F*(1,29)=1.66, *p*=.21; stim*group: *F*(1,29)=.56, *p*=.46) and the beginning (stim: *F*(1,29)=.66, *p*=.43; group: *F*(1,29)=1.81, *p*=.19; stim*group: *F*(1,29)=.14, *p*=.71) or end (stim: *F*(1,29)=3.85, *p*=.06, partial *η^2^*=.12; group: *F*(1,29)=2.84, *p*=.10; stim*group: *F*(1,29)=.47, *p*=.50) of extinction on day 2. There was no group difference on CRs as assessed by FPS at test on day 3 (stim: *F*(1,29)=9.04, *p*=.005, partial *η^2^*=.24; group: *F*(1,29)=.02, *p*=.89; stim*group: *F*(1,29)=.63, *p*=.43). Error bars depict standard error of the mean.

**EXPERIMENT 2**


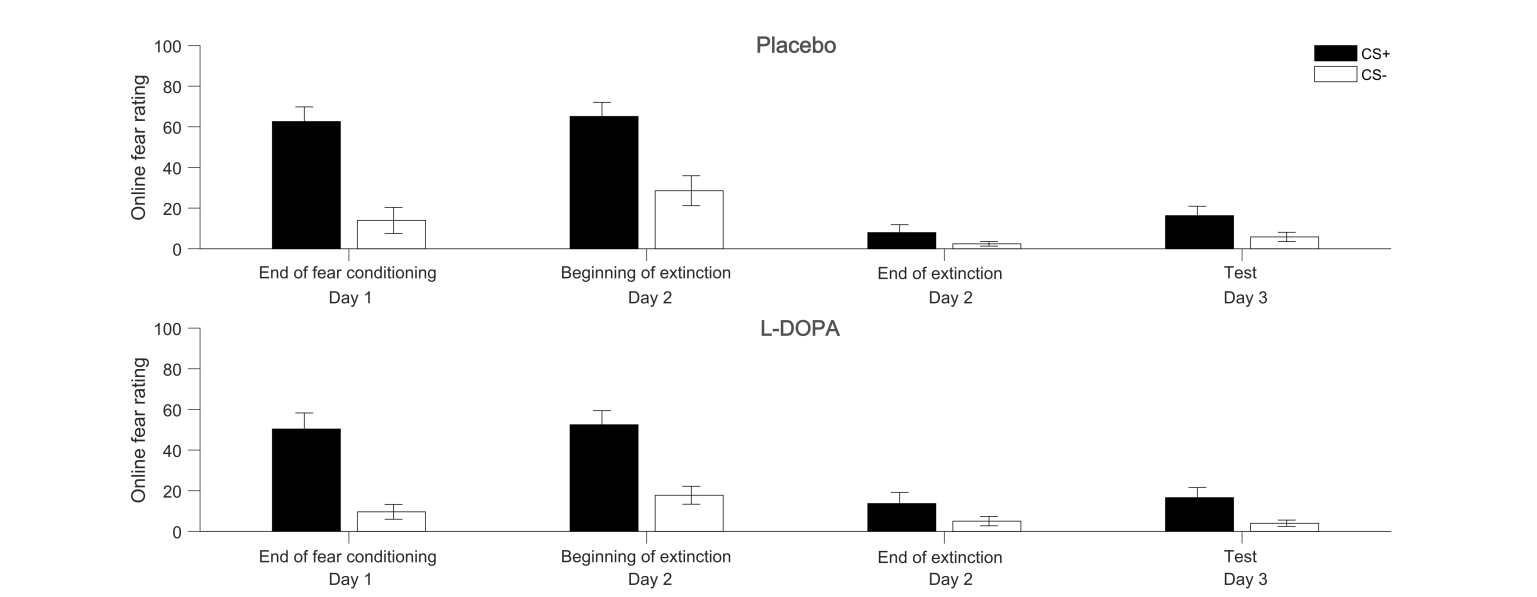


**Supplementary Fig. 5** Mean online fear ratings (0= no fear/tension/distress, 100= maximum fear/tension/distress) during CS+ and CS- presentations in experiment 2 for (a) placebo and (b) L-DOPA treated participants at the end of conditioning on day 1, at the beginning and end of extinction on day 2 and during test on day 3. Repeated measures ANOVA with stimulus (CS+/CS-) as within- and group (placebo/L-DOPA) as between-subject factor revealed no group differences in CRs as assessed by online fear ratings at the end of conditioning on day 1 (stim: *F*(1,30)=71.28, *p*<.001, partial *η^2^*=.70; group: *F*(1,30)=.1.22, *p*=.28; stim*group: *F*(1,30)=.56, *p*=.46) and the beginning (stim: *F*(1,30)=36.59, *p*<.001, partial *η^2^*=.55; group: *F*(1,30)=.33, *p*=.57; stim*group: *F*(1,30)=.16, *p*=.69) or end (stim: *F*(1,30)=5.05, *p*=.03, partial *η^2^*=.14; group: *F*(1,30)=1.11, *p*=.30; stim*group: *F*(1,30)=.25, *p*=.62) of extinction on day 2. There was no effect of L-DOPA administration on online fear ratings during test on day 3 (stim: *F*(1,30)=12.79, *p*=.001, partial *η^2^*=.30; group: *F*(1,30)=.04, *p*=.85; stim*group: *F*(1,30)=.11, *p*=.74). Error bars depict standard error of the mean.

**
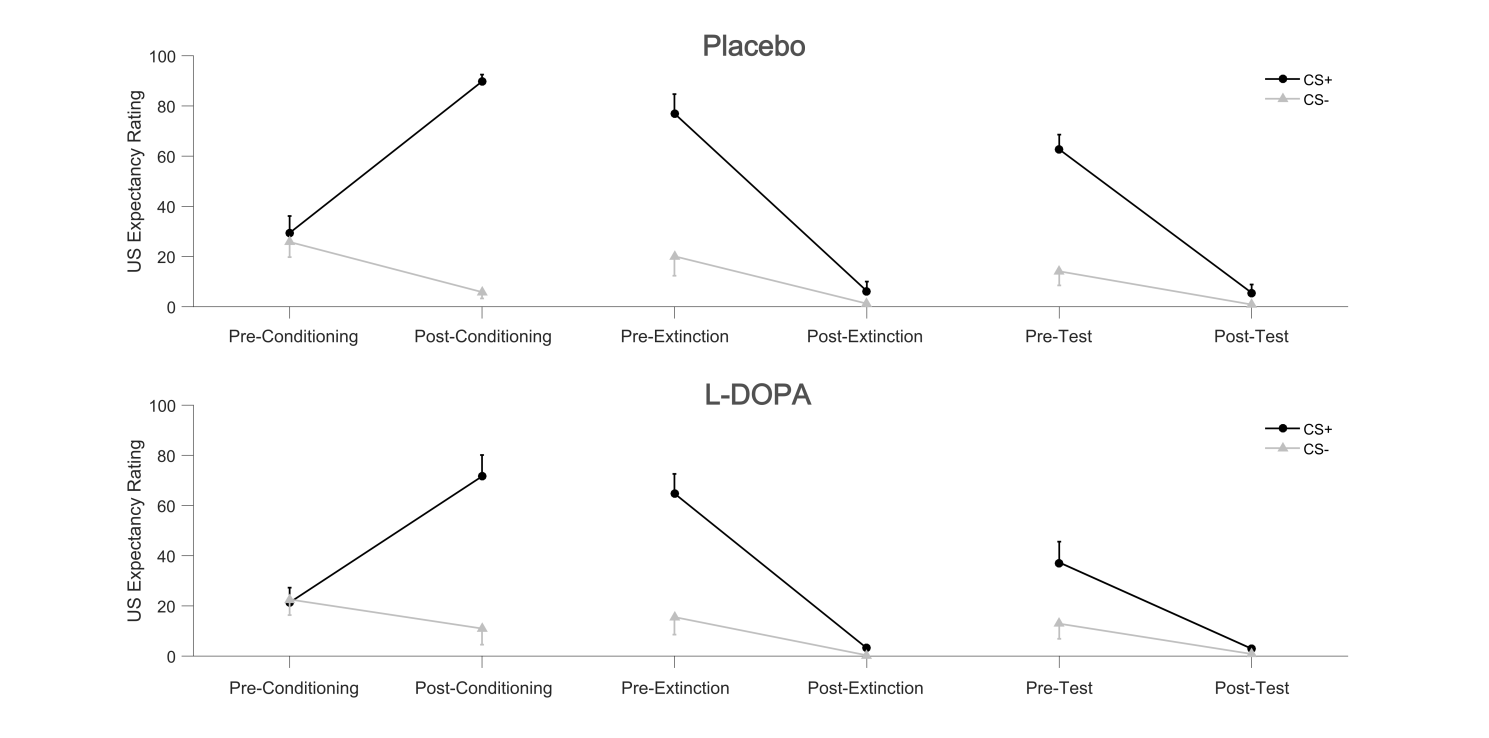
 Supplementary Fig. 6** Mean US expectancy ratings to CS+ and CS- in experiment 1 for (a) placebo and (b) L-DOPA treated participants collected before and after each experimental phase. Repeated measures ANOVA with stimulus (CS+/CS-) as within- and group (placebo/L-DOPA) as between-subject factor revealed no group differences in US expectancy ratings after conditioning on day 1 (stim: *F*(1,30)=115.00, *p*<.001, partial *η^2^*=.79; group: *F*(1,30)=2.29, *p*=.14; stim*group: *F*(1,30)=2.98, *p*=.09) or before (stim: *F*(1,30)=29.96, *p*<.001, partial *η^2^*=.50; group: *F*(1,30)=3.36, *p*=.08; stim*group: *F*(1,30)=.16, *p*=.70) or after (stim: *F*(1,30)=4.16, *p*=.05; group: *F*(1,30)=.83, *p*=.37; stim*group: *F*(1,30)=.31, *p*=.58) extinction on day 2. There was no significant group difference in US expectancy ratings before (stim: *F*(1,30)=40.68, *p*<.001, partial *η^2^*=.58; group: *F*(1,30)=3.76, *p*=.06; stim*group: *F*(1,30)=3.89, *p*=.06) or after test on day 3 (stim: *F*(1,30)=4.01, *p*=.05; group: *F*(1,30)=.43, *p*=.52; stim*group: *F*(1,30)=.59, *p*=.45). Error bars depict standard error of the mean.

**
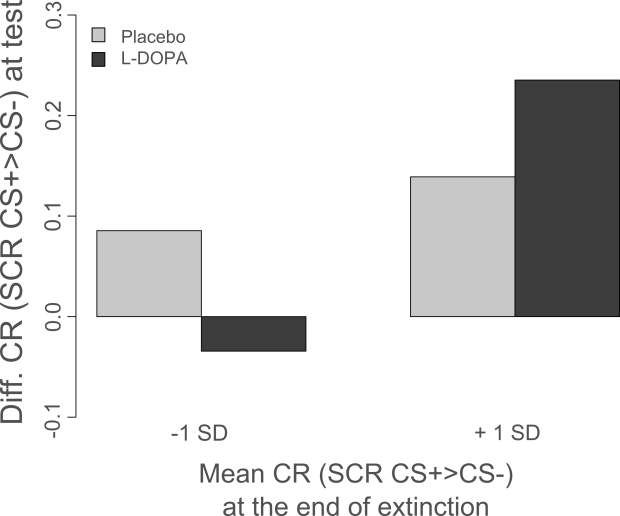
**

**Supplementary Figure 7.** Model-based point estimates of CRs at test on day 3 for placebo and L-DOPA treated participants with low and high CRs at the end of extinction in experiment 1. In line with the results in sub-groups with successful and non-successful extinction (see Figure 3c) also model-based point-estimates of CRs (SCR CS+>CS-) at test on day 3 show that after successful extinction (CR at the end of extinction: mean -1SD) differential CRs at test on day 3 were significantly lower in L-DOPA than in placebo treated participants (β_group_=-.12, SE=.05, *t*(64)=-2.21, *p*=.03). After non-successful extinction (CR at the end of extinction: mean +1SD) CRs at test appeared higher in L-DOPA than in placebo treated participants, even though, the effect was not significant (β_group_=-.10, SE=.06, *t*(64)=1.72, *p*=.09).
